# Lipidic Plaque Features of Polyvascular Disease in Response to Low‐Density Lipoprotein Cholesterol <55 mg/dL: Implication of Circulating Lipoprotein(a) Levels

**DOI:** 10.1161/JAHA.125.048878

**Published:** 2026-08-30

**Authors:** Aya Katasako‐Yabumoto, Yu Kataoka, Stephen J. Nicholls, Eri Kiyoshige, Kunihiro Nishimura, Rishi Puri, Kota Murai, Takamasa Iwai, Kenichiro Sawada, Hideo Matama, Satoshi Honda, Kensuke Takagi, Masashi Fujino, Shuichi Yoneda, Fumiyuki Otsuka, Kazuhiro Nakao, Kensaku Nishihira, Itaru Takamisawa, Yasuhide Asaumi, Kenichi Tsujita, Teruo Noguchi

**Affiliations:** ^1^ Department of Cardiovascular Medicine National Cerebral and Cardiovascular Center Suita, Osaka Japan; ^2^ Department of Advanced Cardiovascular Medicine Graduate School of Medical Sciences, Kumamoto University Kumamoto Japan; ^3^ Victorian Heart Institute (VHI), Medicine Monash Health Clayton VIC Australia; ^4^ Department of Preventive Medicine and Epidemiology National Cerebral and Cardiovascular Center Osaka Japan; ^5^ Department of Cardiovascular medicine Cleveland Clinic Cleveland OH USA; ^6^ Department of Cardiology Miyazaki Medical Association Hospital Miyazaki Japan; ^7^ Department of Cardiology Sakakibara Heart Institute Tokyo Japan; ^8^ Department of Cardiovascular Medicine Graduate School of Medical Sciences, Kumamoto University Kumamoto Japan

**Keywords:** coronary, lipid, low‐density lipoprotein cholesterol, near‐infrared spectroscopy, plaque, polyvascular disease, Vascular Disease, Atherosclerosis, Imaging, Secondary Prevention

## Abstract

**Background:**

Patients with polyvascular disease (PolyVD) or atherosclerosis in the coronaries and at least 1 additional arterial bed are at increased risk of subsequent clinical cardiovascular events. While lowering low‐density lipoprotein cholesterol (LDL‐C) is recommended in the secondary prevention settings, whether PolyVD favorably responds to LDL‐C control remains unknown.

**Methods:**

The current study analyzed in 422 patients (422 target lesions) with coronary artery disease by using near‐infrared spectroscopy imaging before percutaneous coronary intervention in the REASSURE‐NIRS (Revelation of Pathophysiological Phenotypes of Vulnerable Lipid‐Rich Plaque on Near‐Infrared Spectroscopy; NCT04864171) multicenter registry. PolyVD was defined as concomitance of coronary artery disease and cerebrovascular disease or lower extremity arterial disease.

**Results:**

Maximum 4‐mm lipid‐core burden index, as a near‐infrared spectroscopy–derived marker of plaque instability, and lipoprotein(a) [Lp(a)] were compared across LDL‐C levels (<55, 55–69, and ≥70mg/dL) in patients with (n=189) and without PolyVD (n=233). The proportion of LDL‐C <55 mg/dL was 15.3 and 9.4% in patients with and without PolyVD, respectively. In patients without PolyVD, LDL‐C <55 mg/dL was associated with lower maximum 4‐mm lipid‐core burden index (*P*=0.045). Furthermore, a lower frequency of Lp(a) ≥50 mg/dL was observed in patients without PolyVD who achieved LDL‐C <55 mg/dL (*P*=0.042). Only 4.5% of them presented with Lp(a) ≥50 mg/dL. However, achieved LDL‐C <55 mg/dL was not associated with lower maximum 4‐mm lipid‐core burden index in patients with PolyVD (*P*=0.085). Moreover, Lp(a) remained comparable regardless of LDL‐C levels (*P*=0.902). Of note, 20.7% of patients with PolyVD with LDL‐C <55 mg/dL exhibited Lp(a) ≥50 mg/dL.

**Conclusions:**

Coronary atheroma in patients with PolyVD was resistant to lowering LDL‐C <55 mg/dL. Given that >20% of those with LDL‐C <55 mg/dL exhibited Lp(a) ≥50 mg/dL, Lp(a) may be a potential therapeutic target to delipidate coronary atheroma.

## Graphical abstract

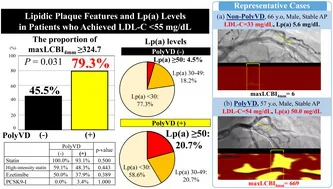

**Nonstandard Abbreviations and Acronyms**
COCOMO‐ACS: Colchicine for Coronary Plaque Modification in Acute Coronary Syndrome
COLCOT: Colchicine Cardiovascular Outcomes Trial
COLOCT: Colchicine for the Stability of Coronary Plaque in Acute Coronary Syndrome
FOURIER: Further Cardiovascular Outcomes Research With PCSK9 Inhibition in Subjects With Elevated Risk
LEAD: lower‐extremity arterial disease
LoDoCo2: Low‐Dose Colchicine 2
maxLCBI_4mm_
: maximum 4‐mm lipid‐core burden index
NIRS: near‐infrared spectroscopy
ODYSSEY: Alirocumab and Cardiovascular Outcomes After Acute Coronary Syndrome
ODYSSEY OUTCOMES: Evaluation of Cardiovascular Outcomes After an Acute Coronary Syndrome During Treatment With Alirocumab
PACMAN‐AMI: Effects of the PCSK9 Antibody Alirocumab on Coronary Atherosclerosis in Patients With Acute Myocardial Infarction
REASSURE‐NIRS: Revelation of Pathophysiological Phenotypes of Vulnerable Lipid‐Rich Plaque on Near‐Infrared Spectroscopy
PolyVD: polyvascular disease

**Clinical Perspective**

**What Is New?**

- Stringent LDL‐C control (LDL‐C <55 mg/dL) was associated with lower maximum 4‐mm lipid‐core burden index and smaller frequency of maximum 4‐mm lipid‐core burden index ≥324.7 in patients without PolyVD, but these benefits were not observed in those with PolyVD.
- Lower circulating Lp(a) concentrations were associated with lower LDL‐C levels in patients without PolyVD. Conversely, among those with PolyVD, Lp(a) levels did not differ significantly across LDL‐C strata (<55, 55–69, and ≥70 mg/dL).

**What Are the Clinical Implications?**

- The present findings highlight the need to identify additional drivers of residual cardiovascular risk in patients with PolyVD, with circulating Lp(a) representing a potential therapeutic target for modifying coronary atheroma of PolyVD.

Polyvascular disease (PolyVD) is a high‐risk phenotype associated with an increased risk of atherosclerotic cardiovascular events. Subanalyses in the FOURIER (Further Cardiovascular Outcomes Research With PCSK9 Inhibition in Subjects With Elevated Risk) and ODYSSEY (Alirocumab and Cardiovascular Outcomes After Acute Coronary Syndrome) studies revealed that PolyVD still continued to present an elevated risk of subsequent atherosclerotic cardiovascular events despite intensive lipid‐lowering therapies.^1^, ^2^ These findings suggest the need for further intensified antiatherosclerotic management in patients with PolyVD.

Recent guidelines from the European Society of Cardiology recommend achieving low‐density lipoprotein cholesterol (LDL‐C) levels <55 mg/dL in patients with atherosclerotic cardiovascular disease (ASCVD).^3^, ^4^ This is based on recent accumulative evidence about favorable efficacy of achieving low LDL‐C levels with potent lipid‐lowering therapies on atherosclerotic plaques and cardiovascular outcomes in patients with ASCVD.^5^, ^6^, ^7^, ^8^, ^9^, ^10^ However, whether this stricter LDL‐C goal provides antiatherosclerotic benefits in patients with PolyVD has not been fully investigated yet.

Lipoprotein(a) [Lp(a)] is a proatherogenic lipoprotein that induces atherogenesis, activation of inflammatory responses, and thrombosis.^11^, ^12^, ^13^ Several published studies showed the association of circulating Lp(a) levels with PolyVD.^14^, ^15^ In particular, Lp(a) levels ≥50 mg/dL was associated with an increased number of involved vessel territories.^14^ While these findings suggest the potential role of Lp(a) in the setting of PolyVD, it remains unknown how this atherogenic lipoprotein affects coronary atheroma in patients with PolyVD who achieved LDL‐C <55 mg/dL.

Near‐infrared spectroscopy imaging (NIRS) enables us to quantitatively evaluate lipidic components within coronary atheroma in vivo.^16^, ^17^, ^18^, ^19^ Therefore, the current study sought to investigate lipidic plaque features in association with achieving LDL‐C <55 mg/dL in patients with and without PolyVD, respectively.

## Methods

### Data Availability

The data that support the findings of this study are available from the corresponding author upon reasonable request, subject to institutional and ethical restrictions.

### Study Subjects

The REASSURE‐NIRS (Revelation of Pathophysiological Phenotypes of Vulnerable Lipid‐Rich Plaque on Near‐Infrared Spectroscopy; NCT04864171) registry is a prospective, multicenter registry cohort that continues to enroll patients with coronary artery disease (CAD) who were imaged by NIRS before percutaneous coronary intervention (PCI). A total of 822 patients were enrolled from August 19, 2015, to June 27, 2025. Of these, 42 patients with restenosis lesions, 10 patients without clinical data, 22 patients without NIRS images before PCI, and 326 patients without any evaluation of extracoronary arteries were excluded. The remaining 422 patients with 422 de novo target lesions who received PCI were analyzed (Figure S1–S4). The research protocol of this study was approved by the institutional review board of the National Cerebral and Cardiovascular Center (M30‐084‐4), the Miyazaki Medical Association Hospital (2020–43), and the Sakakibara Heart Institute (21–007). Written informed consent was not obtained from each patient because this study consisted of an observational analysis of hospitalized patients.

### Definition of PolyVD

In the current study analyzing patients with CAD, PolyVD was defined as the presence of coexisting atherosclerotic extracoronary artery diseases including cerebrovascular disease (CVD) and lower‐extremity arterial disease (LEAD). CVD should have fulfilled at least 1 of the following criteria: a history of stroke or transient ischemic attack, a history of revascularization, the presence of >50% stenosis by the North American Symptomatic Carotid Endarterectomy Trial on duplex ultrasonography. LEAD was defined as the presence of intermittent claudication, an ankle–brachial index <0.9, stenosis of peripheral arteries with its diameter stenosis >50% on angiography or ultrasonography or a history of revascularization. In all patients without any symptoms attributable to extracoronary atherosclerosis, both carotid and lower‐extremity arteries were evaluated by carotid duplex ultrasound or brain magnetic resonance imaging, and ankle–brachial index.

### Acquisition and Quantitative Analysis of NIRS Imaging at Target Lesions

Target lesions receiving PCI were evaluated by NIRS/intravascular ultrasound imaging as reported previously.^20^ In brief, after intracoronary administration of nitroglycerin (100–300 μg), the imaging catheter (TVC Insight or Dualpro; InfraReDx, Bedford, MA) was automatically withdrawn from the far distal site of the target artery (TVC Insight: a translation velocity of 0.5 mm/s and 960 rpm, Dualpro: 2.0 mm/s and 1800 rpm).^21^ Chemogram data in NIRS imaging were quantitatively analyzed with the Makoto system (Infraredx) by physicians (S.K., T.I., K.M., and Y.K.). Each analyst was blinded to the clinical characteristics of the patients. Quantitative analysis was performed to evaluate the amount of lipidic plaque components at target lesions. The probability of lipid cores on the NIRS images was automatically mapped on a red to yellow color scale for visualization. The maximum 4‐mm lipid‐core burden index (maxLCBI_4mm_) was calculated as the number of yellow pixels within target lesions divided by the total number of pixels within the respective segments.^17^, ^18^ The frequency of maxLCBI_4mm_ ≥324.7 was also analyzed.^22^

### Quantitative Coronary Angiography Analysis

Quantitative coronary angiography analysis (reference vessel diameter, minimal lumen diameter, and percentage diameter stenosis) was measured at target lesions by offline commercially available software (QAngio XA; Medis, Leiden, the Netherlands).

### Measurement of Lipid Parameters

Fasting serum levels of Lp(a), triglycerides and high‐density lipoprotein cholesterol were measured by enzymatic methods (Sekisui Medical, Tokyo, Japan) using an automated analyzer (Hitachi Labospect 008; Hitachi‐Hitec, Tokyo, Japan). LDL‐C levels were calculated by the Friedewald formula, except for triglyceride levels >400 mg/dL.^23^ High‐intensity statin was defined as either atorvastatin ≥20 mg, rosuvastatin ≥10 mg, or pitavastatin ≥4 mg according to the guideline of the Japanese Circulation Society and the Japan Atherosclerosis Society.^23^, ^24^

### Statistical Analysis

Categorical values are reported as proportions and were compared using the χ^2^ test or Fisher's exact test, as appropriate. Continuous data are presented as medians with interquartile ranges and were compared using the Kruskal–Wallis test. Comparisons of continuous maxLCBI_4mm_ and Lp(a) were analyzed using linear regression. The proportion of maxLCBI_4mm_ ≥324.7 and Lp(a) ≥50 was analyzed using a general linear model. MaxLCBI_4mm_ levels, the proportion of maxLCBI_4mm_ ≥324.7, Lp(a) levels, and the proportion of Lp(a) ≥50 mg/dL were compared using ANCOVA with adjustment for age, sex, hypertension, type 2 diabetes, current smoker, chronic kidney disease (CKD), acute coronary syndrome (ACS), and high‐intensity statin. One‐to‐one propensity score matching between patients with and without PolyVD was performed to control for potentially confounding variables. Propensity scores were estimated using a multivariable logistic regression model, and 1:1 matching was performed (Methods S1). The variables included age, women, body mass index, type 2 diabetes, CKD, ACS, and high‐intensity statin use. Matching was considered successful when absolute standardized differences were <0.1, indicating good balance (Figure S2 and Table S1–S4). *P* values of <0.05 were significantly different. Statistical analyses were performed with SPSS Statistics version 29.0 for Mac OS (IBM, Armonk, NY).

## Results

### Comparison of Clinical Demographics and NIRS‐Derived Measures Between Patients With and Without PolyVD

The current study included 189 and 233 patients with and without PolyVD, respectively (Figure S1). In patients with PolyVD, the frequencies of CVD, LEAD, and both were 149 (78.8%), 69 (36.5%), and 29 (15.3%), respectively (Table 1). Clinical characteristics were compared in patients with and without PolyVD (Table 1). Patients with PolyVD were more likely to be older (75.0 versus 67.0 years, *P*<0.001) and have a history of hypertension (84.7 versus 72.1%, *P*=0.002) and CKD (62.4 versus 38.2%, *P* < 0.001). Furthermore, lower LDL‐C levels (median, 83.0 [interquartile range, 61.5–105.0] versus 95.0 [interquartile range, 70.5–124.0] mg/dL; *P*<0.001) were observed in patients with PolyVD (Table 1). The proportion of achieving LDL‐C <55 mg/dL was 15.3 and 9.4% in patients with and without PolyVD, respectively (*P*=0.064) (Table 1).

**Table 1:** Characteristics in Patients With and Without PolyVD

	Overall (n=422)	PolyVD (−) (n=233)	PolyVD (+) (n=189)	*P* value
Age, y	71.0 (62.0–77.0)	67.0 (58.0–74.0)	75.0 (68.5–79.0)	<0.001
Women	82 (19.4)	48 (20.6)	34 (18.0)	0.500
BMI, kg/m^2^	23.9 (21.8–25.8)	24.1 (21.9–26.3)	23.8 (21.7–25.3)	0.118
Hypertension	328 (77.7)	168 (72.1)	160 (84.7)	0.002
Dyslipidemia	352 (83.4)	200 (85.8)	152 (80.4)	0.137
Type 2 diabetes	212 (50.2)	113 (48.5)	99 (52.4)	0.428
Current smoker	73 (17.3)	37 (15.9)	36 (19.0)	0.392
Family history	105 (24.9)	69 (29.6)	36 (19.0)	0.013
CKD	207 (49.1)	89 (38.2)	118 (62.4)	<0.001
History of myocardial infarction	100 (23.7)	59 (25.3)	41 (21.7)	0.383
Diagnosis of CAD				
ACS	143 (33.9)	84 (36.1)	59 (31.2)	0.297
Stable CAD	279 (66.1)	149 (63.9)	130 (68.8)	
Extracoronary artery diseases				
Cardiovascular disease	149 (35.3)	…	149 (78.8)	…
Stroke	105 (24.8)	…	105 (55.6)	…
Transient ischemic attack	10 (2.3)	…	10 (5.3)	…
History of carotid revascularization	15 (3.5)	…	15 (7.9)	…
Carotid artery stenosis	49 (11.6)	…	49 (25.9)	…
LEAD	69 (16.3)	…	69 (36.5)	…
Ankle–brachial index	38 (9.0)	…	38 (20.1)	…
Stenosis of peripheral arteries	7 (1.6)	…	7 (3.7)	…
History of revascularization for LEAD	27 (6.3)	…	27 (14.3)	…
Both CVD and LEAD	29 (6.8)	…	29 (15.3)	…
Medication use on admission				
Statin	315 (74.6)	172 (73.8)	143 (75.7)	0.665
High‐intensity statin	142 (33.6)	80 (34.3)	62 (32.8)	0.741
Ezetimibe	103 (24.4)	56 (24.0)	47 (24.9)	0.843
PCSK9‐I	2 (0.5)	1 (0.4)	1 (0.5)	1.000
Aspirin	395 (93.6)	220 (94.4)	175 (92.6)	0.445
P2Y_12_ inhibitor	420 (99.5)	231 (99.1)	189 (100.0)	0.504
ACEI/ARB	214 (50.7)	109 (46.8)	105 (55.6)	0.073
β blocker	175 (41.5)	98 (42.1)	77 (40.7)	0.784
SGLT2i	26 (6.2)	15 (6.4)	11 (5.8)	0.793
Glucagon‐like peptide‐1 inhibitor	28 (6.6)	12 (5.2)	16 (8.5)	0.174
Metformin	60 (14.2)	36 (15.5)	24 (12.7)	0.421
Risk factor control				
LDL‐C, mg/dL	87.0 (65.0–115.0)	95.0 (70.5–124.0)	83.0 (61.5–105.0)	<0.001
LDL‐C <55 mg/dL	51 (12.1)	22 (9.4)	29 (15.3)	0.064
High‐density lipoprotein cholesterol, mg/dL	45.5 (38.0–55.0)	46.0 (39.0–56.0)	45.0 (37.0–53.0)	0.476
Triglyceride, mg/dL	125.5 (87.0–174.2)	130.0 (87.5–179.5)	121.0 (84.0–165.5)	0.438
Glycated hemoglobin, %	6.2 (5.8–6.9)	6.2 (5.8–7.0)	6.2 (5.7–6.8)	0.348
NIRS‐derived measures				
maxLCBI_4mm_	507.5 (343.7–712.0)	533.0 (343.5–708.5)	460.0 (345.0–721.0)	0.409
maxLCBI_4mm_ ≥324.7	327 (77.5)	182 (78.1)	145 (76.7)	0.734

Values are n (%) or median (interquartile range). ACEI indicates angiotensin‐converting enzyme inhibitor; ACS, acute coronary syndrome; ARB, angiotensin II receptor blocker; BMI, body mass index; CAD, coronary artery disease; CKD, chronic kidney disease; CVD, cerebrovascular disease, LEAD, lower‐extremity artery disease, LDL‐C, low‐density lipoprotein cholesterol; maxLCBI_4mm_, maximum 4‐mm lipid‐core burden index; NIRS, near‐infrared spectroscopy; PCSK9‐I, proprotein convertase subtilisin/kexin type 9 inhibitor; PolyVD, polyvascular disease; and SGLT2i, sodium–glucose cotransporter 2 inhibitor.

### Characteristics of PolyVD With and Without Symptoms Attributable to Extracoronary Artery Diseases

In patients with PolyVD (n=189), 121 (64.0%) exhibited symptoms attributable to extracoronary artery diseases, and the remaining 68 (36.0%) did not (Figure 1). In those without symptoms attributable to extracoronary artery diseases (n=68), CVD and LEAD concomitantly existed in 42 and 23 patients, respectively. Both CVD and LEAD were observed in the remaining 3 patients (Figure 1). Table 2 summarizes the comparison of patients with PolyVD with and without symptoms attributable to extracoronary artery diseases. Patients without symptoms attributable to extracoronary artery diseases were more likely to be women (30.9 versus 10.7%, *P*<0.001), and lower proportion of patients with hypertension and statin use (75.0% versus 90.1%, *P*=0.006; 64.7% versus 81.8%, *P*=0.009, respectively) (Table 2). Regardless of symptomatic status, maxLCBI_4mm_ ≥324.7 was observed in >75% of both groups (*P*=0.766) (Table 2).

**Figure 1:**
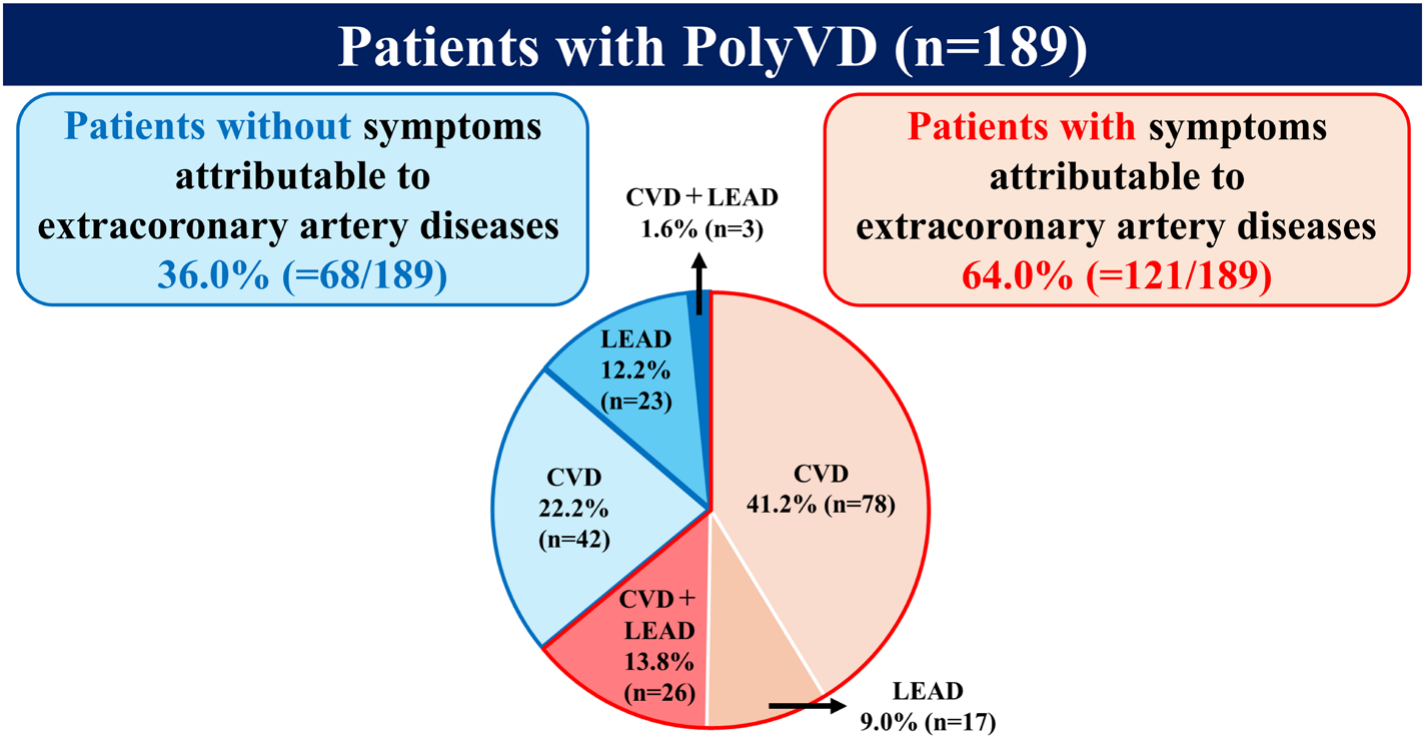
Symptomatic status attributable to extracoronary arteries in patients with PolyVD. CVD indicates cerebrovascular disease; LEAD, lower‐extremity arterial disease; and PolyVD, polyvascular disease.

**Table 2:** Characteristics of PolyVD With and Without Symptoms Attributable to Extracoronary Artery Diseases

	Patients with PolyVD (n=189)		
	Symptoms attributable to extracoronary arteries		
	(−) (n=68)	(+) (n=121)	*P* value
Age, y	76.0 (69.0–79.7)	74.0 (67.5–79.0)	0.322
Women	21 (30.9)	13 (10.7)	<0.001
BMI, kg/m^2^	23.6 (21.9–25.1)	23.8 (21.1–25.3)	0.875
Hypertension	51 (75.0)	109 (90.1)	0.006
Dyslipidemia	50 (73.5)	102 (84.3)	0.073
Type 2 diabetes	36 (52.9)	63 (52.1)	0.908
Current smoker	10 (14.7)	26 (21.5)	0.254
Family history	11 (16.2)	25 (20.7)	0.451
CKD	40 (58.8)	78 (64.5)	0.442
History of myocardial infarction	14 (20.6)	27 (22.3)	0.782
Diagnosis of CAD			
ACS	27 (39.7)	32 (26.4)	0.059
Stable CAD	41 (60.3)	89 (73.6)	
Extracoronary artery diseases			
CVD	45 (66.2)	104 (86.0)	0.001
Stroke	25 (36.8)	80 (66.1)	<0.001
Transient ischemic attack	0 (0)	10 (8.3)	0.015
History of carotid revascularization	0 (0)	15 (12.4)	0.001
Carotid artery stenosis	22 (32.4)	27 (22.3)	0.131
LEAD	26 (38.2)	43 (35.5)	0.712
Ankle–brachial index	24 (35.3)	14 (11.6)	<0.001
Stenosis of peripheral arteries	1 (1.5)	6 (5.0)	0.425
History of revascularization for LEAD	0 (0)	27 (22.3)	<0.001
CVD and LEAD	3 (4.4)	26 (21.5)	0.001
Medication use			
Statin	44 (64.7)	99 (81.8)	0.009
High‐intensity statin	20 (29.4)	42 (34.7)	0.456
Ezetimibe	14 (20.6)	33 (27.3)	0.308
PCSK9‐I	0 (0)	1 (0.8)	1.000
SGLT2i	5 (7.4)	6 (5.0)	0.529
Glucagon‐like peptide‐1 inhibitor, n (%)	8 (11.8)	8 (6.6)	0.222
Metformin	9 (13.2)	15 (12.4)	0.868
Risk factor control			
LDL‐C, mg/dL	83.0 (62.5–118.2)	83.0 (61.0–103.0)	0.442
High‐density lipoprotein cholesterol, mg/dL	45.5 (38.2–55.0)	45.0 (37.0–53.0)	0.488
Triglyceride, mg/dL	115.5 (83.5–164.2)	128.0 (85.5–168.5)	0.435
Glycated hemoglobin, %	6.2 (5.7–6.6)	6.3 (5.8–6.8)	0.452
NIRS imaging			
maxLCBI_4mm_	479.5 (351.5–732.7)	459.0 (338.5–689.5)	0.618
maxLCBI_4mm_ ≥324.7	53 (77.9)	92 (76.0)	0.766

Values are n (%) or median (interquartile range).

ACS indicates acute coronary syndrome; BMI, body mass index; CAD, coronary artery disease; CKD, chronic kidney disease; CVD, cerebrovascular disease; LEAD, lower‐extremity artery disease; LDL‐C, low‐density lipoprotein cholesterol; maxLCBI_4mm_, maximum 4‐mm lipid‐core burden index; NIRS, near‐infrared spectroscopy; PCSK9‐I, proprotein convertase subtilisin/kexin type 9 inhibitor; PolyVD, polyvascular disease; and SGLT2i, sodium–glucose cotransporter 2 inhibitor.

### Comparison of Clinical Characteristics in Patients With PolyVD Diagnosed by History or Vascular Testing

Among the 189 patients with PolyVD, 95 (50.3%) were identified on the basis of clinical history, whereas the remaining 94 (49.7%) had no such history and were diagnosed by vascular testing (Table S2). As shown in Table S2, patients diagnosed by history alone were more likely to exhibit hypertension (95.8% versus 73.4%, *P*<0.001) and to receive statin (82.1% versus 69.1%, *P*=0.038), whereas they were less likely to present with ACS (23.2% versus 39.4%, *P*=0.016). On NIRS imaging analysis, there were no significant differences in maxLCBI_4mm_ (*P*=0.792) and the proportion of maxLCBI_4mm_ ≥324.7 (*P*=0.466) between patients with history‐based and vascular testing–based PolyVD.

### Clinical Demographics in Patients Without PolyVD Stratified According to LDL‐C Levels

The comparison of clinical characteristics is summarized in Table 3. Patients with LDL‐C levels <55 mg/dL were more likely to have a history of myocardial infarction (*P*=0.004) with a lower frequency of ACS (*P*<0.001). Regarding the use of antiatherosclerotic medical therapies, patients with LDL‐C levels 55 mg/dL were more frequently treated with statin (*P*<0.001), high‐intensity statin (*P*=0.002), ezetimibe (*P*<0.001) and β blocker (*P*<0.001). Under these medical therapies, patients with LDL‐C levels <55 mg/dL presented lower levels of triglyceride (*P*=0.021) (Table 3). There were no significant differences in the location of coronary lesions analyzed among 3 groups (*P*=0.310). Coronary lesions in patients with LDL‐C levels <55 mg/dL presented larger minimal lumen diameter (*P*<0.001), accompanied by smaller percentage diameter stenosis (*P*<0.001) (Table 3).

**Table 3:** Comparison of Clinical Characteristics of Patients Without PolyVD Stratified by LDL‐C Categories

	LDL‐C levels			*P* value
	<55 mg/dL (n=22)	55–69 mg/dL (n=34)	≥70 mg/dL (n=177)	
Age, y	68.5 (62.2–77.5)	70.0 (58.7–77.0)	65.0 (57.0–73.0)	0.213
Women	4 (18.2)	7 (20.6)	37 (20.9)	0.957
BMI, kg/m^2^	23.0 (19.8–26.8)	25.0 (21.8–27.1)	24.0 (22.0–25.8)	0.239
Hypertension	18 (81.8)	26 (76.5)	124 (70.1)	0.422
Dyslipidemia	20 (90.9)	30 (88.2)	150 (84.7)	0.670
Type 2 diabetes	11 (50.0)	16 (47.1)	86 (48.6)	0.976
Current smoker	2 (9.1)	1 (2.9)	34 (19.2)	0.039
Family history	3 (13.6)	11 (32.4)	55 (31.1)	0.223
CKD	8 (36.4)	15 (44.1)	66 (37.3)	0.742
History of myocardial infarction	11 (50.0)	12 (35.3)	36 (20.3)	0.004
Diagnosis of CAD				
ACS	1 (4.5)	2 (5.9)	81 (45.8)	<0.001
Stable CAD	21 (95.5)	32 (94.1)	96 (54.2)	
Medication use on admission				
Statin	22 (100.0)	34 (100.0)	116 (65.5)	<0.001
High‐intensity statin	13 (59.1)	17 (50.0)	50 (28.2)	0.002
Ezetimibe	11 (50.0)	15 (44.1)	30 (16.9)	<0.001
PCSK9‐I	0 (0.0)	0 (0.0)	1 (0.6)	0.853
Aspirin	21 (95.5)	31 (91.2)	168 (94.9)	0.668
P2Y_12_ inhibitor	21 (95.5)	34 (100.0)	176 (99.4)	0.136
ACEI/ARB	14 (63.6)	20 (58.8)	75 (42.4)	0.053
β blocker	16 (72.7)	24 (70.6)	58 (32.8)	<0.001
SGLT2i	3 (13.6)	3 (8.8)	9 (5.1)	0.253
Glucagon‐like peptide‐1 inhibitor	0 (0.0)	2 (5.9)	10 (5.6)	0.516
Metformin	4 (18.2)	7 (20.6)	25 (14.1)	0.591
Risk factor control				
LDL‐C, mg/dL	49.0 (43.0–53.0)	63.0 (60.7–65.0)	107.0 (85.0–133.5)	<0.001
High‐density lipoprotein cholesterol, mg/dL	51.5 (42.7–56.2)	41.0 (38.7–55.5)	46.0 (38.0–56.0)	0.255
Triglyceride, mg/dL	101.5 (62.0–131.7)	111.5 (83.7–164.0)	133.0 (89.5–193.5)	0.021
Glycated hemoglobin	6.5 (6.0–6.9)	6.1 (5.6–6.8)	6.2 (5.8–7.1)	0.352
Location of analyzed target lesions				
Left main trunk	0 (0.0)	2 (5.9)	2 (1.1)	0.310
Left anterior descending artery	13 (59.1)	23 (67.6)	101 (57.1)	
Proximal left anterior descending artery	6 (46.1)	13 (56.5)	39 (38.6)	
Left circumflex artery	1 (4.5)	3 (8.8)	28 (15.8)	
Proximal left circumflex artery	0 (0.0)	0 (0.0)	7 (25.0)	
Right coronary artery	8 (36.4)	6 (17.6)	45 (25.6)	
Proximal right coronary artery	4 (50.0)	1 (16.6)	16 (35.5)	
Saphenous vein graft	0 (0.0)	0 (0.0)	0 (0.6)	
Quantitative coronary angiography analysis at target lesions				
Reference diameter, mm	3.0 (2.5–3.5)	2.8 (2.2–3.2)	2.8 (2.2–3.1)	0.197
Minimal lumen diameter, mm	1.1 (0.8–1.3)	0.9 (0.6–1.3)	0.6 (0.3–1.0)	<0.001
Diameter stenosis, %	66.7 (52.0–74.2)	65.2 (52.2–76.5)	78.0 (61.9–90.0)	<0.001

Values are n (%) or median (interquartile range). ACEI indicates angiotensin‐converting enzyme inhibitor; ACS, acute coronary syndrome; ARB, angiotensin II receptor blocker; BMI, body mass index; CAD, coronary artery disease; CKD, chronic kidney disease; LDL‐C, low‐density lipoprotein cholesterol, PCSK9‐I, proprotein convertase subtilisin/kexin type 9 inhibitor; PolyVD, polyvascular disease; and SGLT2i, sodium–glucose cotransporter 2 inhibitor.

### LDL‐C Levels <55 mg/dL and MaxLCBI_4mm_ at Target Lesions in Patients Without PolyVD

Figure 2 showed the comparison of maxLCBI_4mm_ at target lesions in 3 groups. In patients without PolyVD, a lower maxLCBI_4mm_ was observed in association with lower LDL‐C levels (*P*=0.045; Figure 2). Of note, in those who achieved LDL‐C <55 mg/dL, maxLCBI_4mm_ was significantly lower than other groups (Figure 2). Furthermore, patients with LDL‐C levels <55 mg/dL more likely exhibited a lower proportion of maxLCBI_4mm_ ≥324.7 (*P*=0.017; Figure 2). In the propensity score–matched cohort, among patients without PolyVD, both log‐transformed maxLCBI_4mm_ (*P*=0.031) and the proportion of maxLCBI_4mm_ ≥324.7 (*P*=0.001) increased across LDL‐C categories (Table S3).

**Figure 2:**
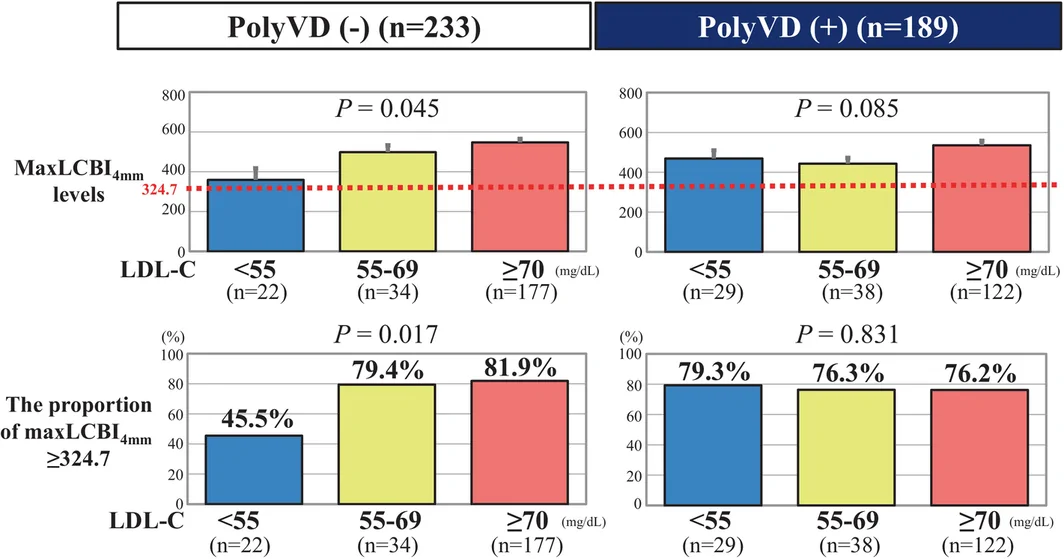
LDL‐C<55 mg/dL and MaxLCBI_4mm_ in patients with and without PolyVD. **A**, MaxLCBI_4mm_ levels. **B**, Proportion of MaxLCBI_4mm_≥324.7. The red dashed line indicates the threshold value of 324.7. LDL‐C indicates low‐density lipoprotein cholesterol; MaxLCBI_4mm_, maximum 4‐mm lipid‐core burden index; and PolyVD, polyvascular disease.

### Clinical Demographics in Patients With PolyVD Stratified According to LDL‐C Levels

Table 4 describes the comparison of clinical demographics in patients with PolyVD stratified according to LDL‐C levels (<55, 55–69, and ≥70 mg/dL). The proportion of those with PolyVD achieving LDL‐C levels <55 mg/dL was 15.3%. Patients with LDL‐C levels <55 mg/dL were more likely to have a history of myocardial infarction (*P*<0.001), whereas the frequency of other coronary risk factors was similar in 3 groups (Table 4). Moreover, there were significant differences in lipid‐lowering therapies (statin, *P*<0.001; high‐intensity statin, *P*<0.001; ezetimibe, *P*=0.001), aspirin (*P*=0.045), and β blocker (*P*=0.010). Lower levels of high‐density lipoprotein cholesterol (*P*=0.008) and triglyceride (*P*=0.021) were observed in patients with LDL‐C levels <55 mg/dL. Similar to patients without PolyVD, larger minimum lumen diameter (*P*=0.010) and smaller percentage diameter stenosis (*P*=0.007) were observed in those with PolyVD who achieved LDL‐C levels <55 mg/dL (Table 4).

**Table 4:** Comparison Clinical and Demographic Characteristics of Patients With PolyVD Stratified by LDL‐C Categories

	LDL‐C levels			*P* value
	<55 mg/dL (n=29)	55–69 mg/dL (n=38)	≥70 mg/dL (n=122)	
Age, y	73.0 (65.5–81.0)	75.0 (66.5–79.0)	74.5 (69.0–79.0)	0.889
Women	4 (13.8)	4 (10.5)	26 (21.3)	0.260
BMI, kg/m^2^	24.3 (21.7–25.5)	23.5 (22.1–26.4)	23.5 (21.4–25.3)	0.423
Hypertension	25 (86.2)	31 (81.6)	104 (85.2)	0.834
Dyslipidemia	24 (82.8)	31 (81.6)	97 (79.5)	0.906
Type 2 diabetes	17 (58.6)	22 (57.9)	60 (49.2)	0.492
Current smoker	2 (6.9)	6 (15.8)	28 (23.0)	0.120
Family history	9 (31.0)	8 (21.1)	19 (15.6)	0.153
CKD	23 (79.3)	21 (55.3)	74 (60.7)	0.104
History of myocardial infarction	12 (41.4)	13 (34.2)	16 (13.1)	<0.001
Diagnosis of CAD				
ACS	7 (24.1)	5 (13.2)	47 (38.5)	0.009
Stable CAD	22 (75.9)	33 (86.8)	75 (61.5)	
Medication use on admission				
Statin	27 (93.1)	37 (97.4)	79 (64.8)	<0.001
High‐intensity statin	14 (48.3)	21 (55.3)	27 (22.1)	<0.001
Ezetimibe	11 (37.9)	16 (42.1)	20 (16.4)	0.001
PCSK9‐I	1 (3.4)	0 (0.0)	0 (0.0)	0.062
Aspirin	26 (89.7)	32 (84.2)	117 (95.9)	0.045
P2Y_12_ inhibitor	29 (100.0)	38 (100.0)	122 (100.0)	N/A
ACEI/ARB	18 (62.1)	22 (57.9)	65 (53.3)	0.657
β blocker	15 (51.7)	22 (57.9)	40 (32.8)	0.010
SGLT2i	2 (6.9)	4 (10.5)	5 (4.1)	0.324
Glucagon‐like peptide‐1 inhibitor	3 (10.3)	4 (10.5)	9 (7.4)	0.768
Metformin	3 (10.3)	5 (13.2)	16 (13.1)	0.918
Risk factor control				
LDL‐C, mg/dL	49.0 (39.5–51.5)	62.5 (58.0–67.0)	98.5 (83.0–120.0)	<0.001
High‐density lipoprotein cholesterol, mg/dL	38.0 (32.5–47.0)	47.0 (40.7–53.0)	46.5 (37.7–55.2)	0.008
Triglyceride, mg/dL	96.0 (72.0–143.5)	106.5 (71.0–161.0)	129.5 (94.0–176.5)	0.021
Glycated hemoglobin, %*	6.3 (5.8–6.7)	6.4 (5.8–6.9)	6.1 (5.7–6.7)	0.606
Location of target lesions				
Left main trunk	0 (0.0)	0 (0.0)	1 (0.8)	0.527
Left anterior descending artery	14 (48.3)	23 (60.5)	72 (59.0)	
Proximal left anterior descending artery	3 (21.4)	7 (30.4)	16 (22.2)	
Left circumflex artery	4 (13.8)	3 (7.9)	21 (17.2)	
Proximal left circumflex artery	2 (50.0)	1 (33.3)	6 (28.5)	
Right coronary artery,	11 (37.9)	12 (31.6)	28 (23.0)	
Proximal right coronary artery	4 (36.3)	2 (16.6)	12 (42.8)	
Quantitative coronary angiography analysis at target lesions				
Reference diameter, mm	2.6 (2.2–3.0)	2.6 (2.3–3.0)	2.6 (2.2–3.1)	0.886
Minimal lumen diameter, mm	0.7 (0.5–1.0)	0.8 (0.5–1.1)	0.5 (0.3–1.0)	0.010
Diameter stenosis, %	69.2 (60.9–80.6)	63.6 (55.6–77.0)	78.3 (61.9–89.9)	0.007

Values are n (%) or median (interquartile range). ACEI indicates angiotensin‐converting enzyme inhibitor; ACS, acute coronary syndrome; ARB, angiotensin II receptor blocker; BMI, body mass index; CAD, coronary artery disease; CKD, chronic kidney disease; HDL‐C, high‐density lipoprotein cholesterol; LDL‐C, low‐density lipoprotein cholesterol; PCSK9‐I, proprotein convertase subtilisin/kexin type 9 inhibitor; PolyVD, polyvascular disease; and SGLT2i, sodium–glucose cotransporter 2 inhibitor.

### LDL‐C Levels <55 mg/dL and MaxLCBI_4mm_ at Target Lesions in Patients with PolyVD

Figure 2 illustrated the comparison of maxLCBI_4mm_ in patients with PolyVD stratified according to LDL‐C levels. In contrast with patients without PolyVD, achieving lower LDL‐C levels was not associated with maxLCBI_4mm_ in patients with PolyVD (*P*=0.085; Figure 2). Moreover, there was no significant difference in the proportion of maxLCBI_4mm_ ≥324.7 in 3 groups (*P* = 0.831; Figure 2). Even under lowering LDL‐C <55 mg/dL, 79.3% of patients with PolyVD continued to present maxLCBI_4mm_ ≥324.7 at their target lesions (Figure 2). Similarly, in the propensity score–matched cohort, neither log‐transformed maxLCBI_4mm_ (*P*=0.481) nor the proportion of maxLCBI_4mm_ ≥324.7 (*P*=0.747) differed significantly across LDL‐C categories among patients with PolyVD (Table S3).

In patients with PolyVD (n=189), 2 and 3 vascular territories were involved in 84.7 (n=160) and 15.3% (n=29) of them, respectively. NIRS‐derived measures were compared across 3 LDL‐C subgroups in association with the number of vascular territories (Figure 3). Although the raw maxLCBI_4mm_ values appeared broadly similar across the 3 groups in patients with 2 vascular territories' involvement, ANCOVA demonstrated a significant difference after adjustment for prespecified covariates (*P*=0.046). In patients with 3 vascular territories' involvement, maxLCBI_4mm_ values were comparable among 3 groups (*P*=0.966). The proportion of maxLCBI_4mm_ **≥**324.7 (2 vascular territories, *P*=0.434; 3 vascular territories, *P*=0.376) did not differ across 3 groups in both patients with 2 and 3 vascular territories' involvement (Figure 3).

**Figure 3:**
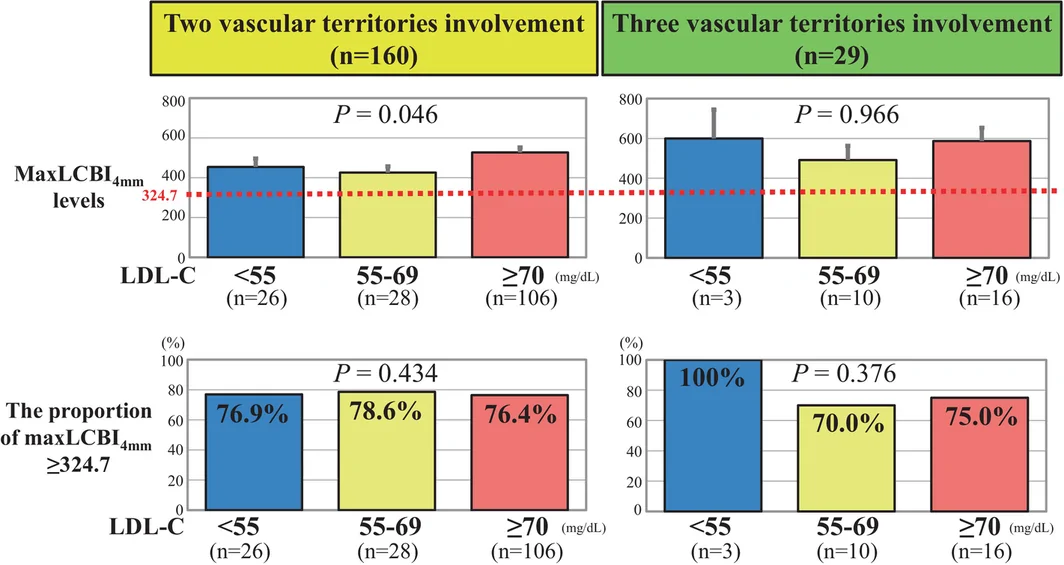
Number of involved extracoronary arteries and NIRS‐derived MaxLCBI_4mm_ levels in patients without PolyVD. The red dashed line indicates the threshold value of 324.7. LDL‐C indicates low‐density lipoprotein cholesterol; MaxLCBI_4mm_, maximum 4‐mm lipid‐core burden index; NIRS, near‐infrared spectroscopy; and PolyVD, polyvascular disease.

### Comparison of MaxLCBI_4mm_ ≥324.7 in Each LDL‐C Subgroup Between Patients With and Without PolyVD

Further comparisons of clinical characteristics and maxLCBI_4mm_ ≥324.7 in each LDL‐C subgroup were conducted between patients with and without PolyVD (Table 5 and Table S4). In patients with LDL‐C <55 mg/dL, a greater frequency of CKD was observed in those with PolyVD (79.3 versus 36.4%, *P* = 0.002) (Table S4), whereas antiatherosclerotic medication use and the degree of risk factor control did not differ between the 2 groups. On NIRS imaging analysis, despite this stringent LDL‐C control, patients with PolyVD still exhibited a greater frequency of maxLCBI_4mm_ ≥324.7 compared with those without PolyVD (79.3 versus 45.5%, *P*=0.031) (Table 5, Graphical Abstract). In patients with LDL‐C between 55 and 69 mg/dL, there were no significant differences in clinical characteristics and the proportion of maxLCBI_4mm_ ≥324.7 (76.3 versus 79.4%, *P*=0.905) between those with and without PolyVD (Table 5 and Table S4). In patients with LDL‐C ≥70 mg/dL, patients with PolyVD were more likely to have a history of hypertension (85.2 versus 70.1%, *P*=0.002) and CKD (60.7 versus 37.3%, *P*<0.001) with a lower prevalence of family history (15.6 versus 31.1%, *P*=0.002). A lower LDL‐C level was observed in those with PolyVD (median, 98.5 [interquartile range, 83.0–120.0] versus 107.0 [interquartile range, 85.0–133.5] mg/dL; *P* = 0.035) (Table S4). However, the prevalence of maxLCBI_4mm_ ≥324.7 did not differ between patients with and without PolyVD (76.2 versus 81.9%, *P*=0.192) (Table 5).

**Table 5:** Comparison of MaxLCBI_4mm_≥324.7 in Each LDL‐C Subgroup

LDL‐C<55 mg/dL (n=51)			
	PolyVD (−) (n=22)	PolyVD (+) (n=29)	*P* value
MaxLCBI_4mm_≥324.7, n (%)	10 (45.5)	23 (79.3)	0.031

LDL‐C 55–69 mg/dL (n=72)			
	PolyVD (−) (n=34)	PolyVD (+) (n=38)	*P* value
MaxLCBI_4mm_≥324.7, n (%)	27 (79.4)	29 (76.3)	0.905

LDL‐C>70 mg/dL (n=299)			
	PolyVD (−) (n=177)	PolyVD (+) (n=122)	*P* value
MaxLCBI_4mm_≥324.7, n (%)	145 (81.9)	93 (76.2)	0.192

MaxLCBI_4mm_ was adjusted for age, sex, hypertension, type 2 diabetes, current smoker, CKD, ACS, and high‐intensity statin. ACS indicates acute coronary syndrome; CKD, chronic kidney disease; LDL‐C, low‐density lipoprotein cholesterol; MaxLCBI_4mm_, maximum 4‐mm lipid‐core burden index; and PolyVD, polyvascular disease.

### Lp(a) Levels and the Proportion of Lp(a) ≥50 mg/dL in Patients With and Without PolyVD

In patients with and without PolyVD, Lp(a) levels were compared across 3 LDL‐C subgroups, respectively (Figure 4). In patients without PolyVD, lower Lp(a) levels (*P*=0.033) and a lower frequency of Lp(a) ≥50 mg/dL (*P*=0.042) were observed in those with LDL‐C <55 mg/dL (Figure 4). Only 4.5% of those with LDL‐C <55 mg/dL presented Lp(a) ≥50 mg/dL. By contrast, in those with PolyVD, there were no significant differences in Lp(a) levels (*P*=0.902) and the frequency of Lp(a) ≥50 mg/dL (*P*=0.560) across 3 groups (Figure 4). Of note, >20% of patients with PolyVD who achieved LDL‐C <55 mg/dL exhibited Lp(a) ≥50 mg/dL.

**Figure 4:**
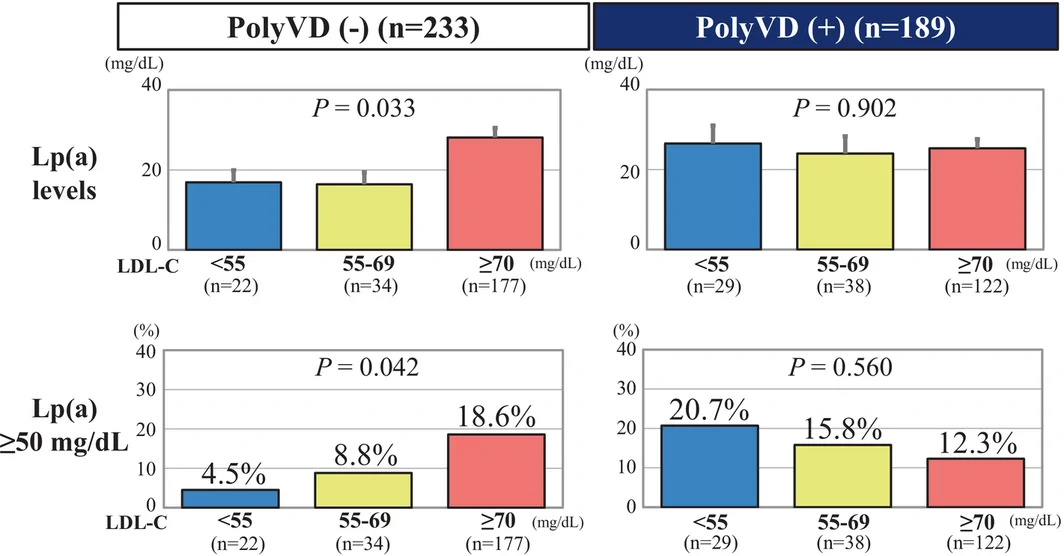
Lp(a) in patients with and without PolyVD stratified by LDL‐C levels. **A**, Lp(a) levels. **B**, Proportion of Lp(a) ≥50 mg/dL. LDL‐C indicates low‐density lipoprotein cholesterol; Lp(a), lipoprotein(a); and PolyVD, polyvascular disease.

## Discussion

PolyVD harbors extensive atherosclerotic disease, which is characterized as propagation of atherosclerosis into multiple vascular beds. While stringent control of LDL‐C levels has been shown to favorably modulate atherosclerosis,^7^, ^25^ whether atherosclerotic disease substrate in the setting of PolyVD responds to guideline‐recommended lipid‐lowering therapy remains to be fully elucidated yet. In the current study using NIRS imaging, maxLCBI_4mm_ significantly decreased in association with achieving LDL‐C <55 mg/dL in patients without PolyVD. In patients with PolyVD, however, this favorable association of maxLCBI_4mm_ with LDL‐C levels did not exist. Lipid‐rich plaque feature was still observed in 79% of patients with PolyVD achieving LDL‐C <55 mg/dL. These findings suggest the attenuated efficacy of lowering LDL‐C <55 mg/dL on coronary atherosclerosis in patients with PolyVD.

Our NIRS imaging analysis provides mechanistic insights into worse cardiovascular outcomes in patients with PolyVD. Recent studies consistently demonstrated that lowering LDL‐C levels induced a reduction of maxLCBI_4mm_ in patients with CAD.^5^, ^8^ In the current study, as shown in Figure 2, a lower LDL‐C level was not necessarily associated with an increased frequency of delipidated coronary atherosclerosis. Of note, even under control of LDL‐C <55 mg/dL in patients with PolyVD, only 21% of them presented a stable form of disease. Additionally, the frequency of maxLCBI_4mm_ ≥324.7 was significantly higher in patients with PolyVD compared with those without PolyVD. Since maxLCBI_4mm_ has been shown as a plaque measure to predict future risks of cardiovascular events, these findings indicate that the continuing residual disease of PolyVD despite antiatherosclerotic management could account for their worse cardiovascular outcomes.

Achieving stricter LDL‐C levels provides antiatherosclerotic benefits in patients with ASCVD.^26^ However, our findings suggest that its favorable efficacy was diminished in patients with PolyVD. One of the potential mechanisms behind this observation would be an elevated inflammatory activity in the setting of PolyVD. A recent study reported elevated levels of high‐sensitivity CRP and interleukin‐6 in patients with PolyVD.^27^, ^28^, ^29^ As shown in our analysis, target lesions in patients with PolyVD more likely contained greater lipidic materials despite control of LDL‐C <55 mg/dL. Given that lipid‐rich plaque harbor inflammatory materials, modulating inflammation may be required to further stabilize vulnerable form of disease in patients with PolyVD. Recent randomized controlled trials have demonstrated cardiovascular benefits of colchicine that modulates interleukin‐1β and interleukin‐6.^30^, ^31^ The COLCOT (Colchicine Cardiovascular Outcomes Trial) study has shown that the use of colchicine lowered risks of ASCVD in patients with myocardial infarction.^32^ In the LoDoCo2 (Low‐Dose Colchicine 2) study, patients with chronic coronary syndromes benefited from colchicine to mitigate their subsequent cardiovascular event risks.^33^ Additionally, serial optical coherence tomography imaging studies (COLOCT [Colchicine for the Stability of Coronary Plaque in Acute Coronary Syndrome] and COCOMO‐ACS [Colchicine for Coronary Plaque Modification in Acute Coronary Syndrome] studies) have revealed an increase in fibrous cap thickness following the commencement of colchicine in the setting of ACS.^32^, ^34^, ^35^ According to these observations, the current American College of Cardiology/American Heart Association guideline recommends considering the use of colchicine in patients with ACS (class 2b recommendation).^36^ Since patients with PolyVD are more likely to exhibit elevated levels of interleukin‐6,^29^ colchicine may be an additive agent to modulate inflammation, leading to a reduction in subsequent cardiovascular event risks in patients with PolyVD. Regarding interleukin‐6, novel therapy targeting interleukin‐6 is under investigation in the phase III trials (NCT05636176). Future studies are expected to elucidate whether emerging therapies lowering interleukin‐6 could modify disease and improve cardiovascular outcomes in patients with PolyVD.

Another mechanism may be the proatherogenic properties of circulating Lp(a). Pathophysiologically, Lp(a) presents versatile properties which promote atherosclerosis, thrombosis, and inflammatory activity.^37^, ^38^, ^39^ Recently published studies have reported elevated Lp(a) levels as a residual proatherogenic risk in patients receiving lipid‐lowering therapies. One subanalysis from the PACMAN‐AMI (Effects of the PCSK9 Antibody Alirocumab on Coronary Atherosclerosis in Patients With Acute Myocardial Infarction) trial showed that Lp(a) ≥125 nmol/L (≥50 mg/dL) attenuated the efficacy of alirocumab to modulate maxLCBI_4mm_ compared with Lp(a) <125 nmol/L.^11^ Another subanalysis from the ODYSSEY OUTCOMES (Evaluation of Cardiovascular Outcomes After an Acute Coronary Syndrome During Treatment With Alirocumab) trial focused on placebo group (n=9149) who received 40 to 80 mg of atorvastatin or 20 to 40 mg of rosuvastatin.^13^ In this analysis, baseline Lp(a) levels continuously increased risks of future cardiovascular events independently of LDL‐C. Of note, even in patients with low Lp(a) levels (<21.4 mg/dL), Lp(a) levels were still associated with elevated risk of major adverse cardiovascular events. In addition to these observations, several observational studies showed a higher Lp(a) levels as an independent factor associated with maxLCBI_4mm_ in patients receiving lipid‐lowering therapies.^20^, ^40^ These findings suggest that the degree of maxLCBI_4mm_ could be affected by circulating Lp(a) levels even in achieving lower LDL‐C levels. In our patients without PolyVD, lower Lp(a) levels were observed in those with LDL‐C <55 mg/dL, which may lead to their lower maxLCBI_4mm_. In patients with PolyVD, however, Lp(a) levels did not differ across three LDL‐C subgroups. Given that the proportion of Lp(a) ≥50 mg/dL was >20% of patients with PolyVD who achieved LDL‐C <55 mg/dL, this Lp(a)‐mediated characteristic may support their residual lipidic plaque features. Currently, clinical trials are ongoing to test the efficacy of agents lowering Lp(a). Lepodisiran is a small interfering RNA agent to lower Lp(a) levels by 94%.^41^ Olpasiran and zerlasiran are other small interfering RNAs that lower Lp(a) levels by about 95%.^42^, ^43^ These agents are expected to further reduce risks of future ASCVD events in high‐risk patients. Given the elevated Lp(a) levels in patients with PolyVD, small interfering RNA agents lowering Lp(a) may provide antiatherosclerotic benefits in patients with PolyVD. Future studies are expected to elucidate the efficacy of these novel agents in the setting of PolyVD.

The current study provides insights into characteristics of PolyVD without any symptoms attributable to extracoronary arteries. As shown in Figure 1, 36.0% of PolyVD did not exhibit symptoms driven by extracoronary artery diseases. Since over 30% of patients with PolyVD without any symptoms attributable to extracoronary arteries were women, this may be associated with an undertreated feature of them, reflected by a lower frequency of their statin use. Another intriguing observation is that symptomatic status related to extracoronary arteries did not affect the severity of lipidic plaque materials within coronary atheroma. This finding highlights the importance of intensive antiatherosclerotic management in patients with PolyVD regardless of their extracoronary‐related symptomatic status.

NIRS‐derived lipid‐core burden index is a histologically validated measure associated with plaque instability. One validation study using coronary specimens showed that NIRS‐derived lipid‐core burden index corresponds to the presence of thin cap fibroatheroma.^17^ Moreover, the degree of lipid‐core burden index was associated with the volume of necrotic core materials within fibroatheroma.^17^ These observations indicate that lesions with elevated maxLCBI_4mm_ could exhibit thinner fibrous caps and greater necrotic core volumes, which are associated with plaque instability. Optical coherence tomography imaging is a high‐resolution intravascular imaging modality that enables visualization of fibrous cap and other plaque microstructures in vivo. Optical coherence tomography imaging studies revealed grater frequencies of fibrous cap thickness <75 μm, thin cap fibroatheroma, macrophages, and intraplaque intimal vasculature at coronary lesions exhibiting elevated maxLCBI_4mm_.^44^, ^45^ These findings also support the association of maxLCBI_4mm_ with plaque instability. Given that elevated maxLCBI_4mm_ has been shown to predict future cardiovascular event risk,^22^, ^46^ the existence of elevated maxLCBI_4mm_ despite achieving lower LDL‐C levels in patients with PolyVD suggests their residual cardiovascular risk.

Several caveats should be noted. First, this is an observational study which includes a relatively small number of patients with PolyVD. Second, the use of NIRS imaging and the selection of lipid‐lowering therapies were decided according to each physician's discretion. Therefore, the possibility of selection bias cannot be excluded. Third, the approved dose of high‐intensity statin in Japan is different from that in foreign countries, which may affect our findings. Racial differences in response to statins have been previously reported. One previous study revealed that plasma concentration of rosuvastatin and its metabolites were significantly higher in Japanese patients compared to non‐Japanese ones.^47^ Due to racial differences, the approved maximum doses of atorvastatin and rosuvastatin in Japan are lower than those in Western countries. Current guidelines from the Japanese Circulation Society and Japan Atherosclerosis Society define high‐intensity statins as rosuvastatin 10 to 20 mg, atorvastatin 20 to 40 mg, and pitavastatin 4 mg.^23^, ^48^ Whether the use of larger doses of statins affected our findings remains unknown. Fourth, the REASSURE‐NIRS registry enrolls patients with CAD receiving PCI. Therefore, the current study did not include those with CVD and LEAD but not CAD. Fifth, the current study did not measure inflammatory markers. Sixth, the current study conducted cross‐sectional imaging with NIRS at coronary lesions but not serial imaging. Additionally, NIRS imaging enables evaluation of lipidic plaque contents, but not other morphological features. Therefore, the further detailed plaque characteristics in patients with and without PolyVD under lowering LDL‐C remain unknown. Seventh, statistical power about the comparison of maxLCBI_4mm_ was 99.6% in patients without PolyVD, and 95.8% in those with PolyVD, respectively. Due to the retrospective nature of our analysis, future prospective studies with appropriate statistical power and sample size are needed to further elucidate plaque features in patients with PolyVD in response to LDL‐C lowering.

In conclusion, 15.3% and 9.4% of patients with and without PolyVD, respectively, presented LDL‐C <55 mg/dL at the index of PCI. This strict LDL‐C control was associated with a smaller maxLCBI_4mm_ in patients without PolyVD. However, this benefit was not observed in those with PolyVD. Of note, despite lowering LDL‐C <55 mg/dL, lipid‐rich plaque still existed in 79.3% of patients with PolyVD. Additional analyses revealed that lower levels of circulating Lp(a) were observed in association with lower LDL‐C levels in patients without PolyVD. In contrast, circulating Lp(a) levels was not different across 3 groups of patients with PolyVD regardless of LDL‐C levels. The current findings support the need for finding drivers and targets to achieve further effective reductions in cardiovascular risk of PolyVD. Circulating Lp(a) may be a potential therapeutic target in patients with PolyVD to further modulate their coronary atheroma.

## Sources of Funding

This study was supported by the Fukuda Foundation for Medical Technology.

## Disclosures

Y.K. has received research support from Kowa, Nipro, and Abbott, and honoraria from Nipro, Abbott, Kowa, Amgen, Sanofi, Astellas, Takeda, and Daiichi‐Sankyo. S.J.N. is a recipient of a Principal Research Fellowship from the National Health and Medical Research Council of Australia and has received research support from AstraZeneca, Amgen, Anthera, CSL Behring, Cerenis, Eli Lilly, Esperion, Resverlogix, Novartis, InfraReDx, and Sanofi–Regeneron and is a consultant for Amgen, Akcea, AstraZeneca, Boehringer Ingelheim, CSL Behring, Eli Lilly, Esperion, Kowa, Merck, Takeda, Pfizer, Sanofi–Regeneron and Novo Nordisk. R.P. has received speaker fees from Amgen and Sanofi; served as a consultant for Cerenis, Medtronic, Philips, Boston Scientific, and Shockwave and on advisory boards for Centerline Biomedical, Medtronic, and Bioventrix; and holds minor equity in Centerline Biomedical. The remaining authors have nothing to disclose.

## Supporting information

STROBE Statement—Checklist

Table S1–S4
Figure S1–S2
