# Supplementary material for: Lipidic Plaque Features of Polyvascular Disease in Response to Low‐Density Lipoprotein Cholesterol <55 mg/dL: Implication of Circulating Lipoprotein(a) Levels
Source: J Am Heart Assoc. 2026 Aug 30;15(19):e048878. doi: 10.1161/JAHA.125.048878 (PMC13745112; doi:10.1161/JAHA.125.048878)
Supplement: Supplementary file 2 [file JAH3-15-e048878-s002.pdf]

Supplemental Material

Method S1. Propensity Score–Matched Analysis

To address potential baseline differences between patients with and without polyvascular disease (PolyVD), we performed 1:1 propensity score matching using nearest-neighbor matching based on age, sex, body mass index, diabetes mellitus, chronic kidney disease, acute coronary syndrome, and high-intensity statin use. A caliper width of 0.1 SD was applied. Covariate balance after matching was assessed using standardized mean differences, and a value <0.1 was considered to indicate adequate balance. In the matched cohort, patients were further stratified according to LDL-C category (<55, 55–69, and ≥70 mg/dL) and PolyVD status. For continuous outcomes, $maxLCBI_{4mm}$ was log-transformed before analysis because of their skewed distributions. Differences across LDL-C categories were assessed separately in patients with and without PolyVD using weighted linear regression, and P for trend was calculated by modeling LDL-C category as an ordinal variable. For binary analyses, the proportion of $maxLCBI_{4mm}$ ≥324.7 was compared across LDL-C categories using weighted logistic regression, and P for trend was similarly calculated with LDL-C category entered as an ordinal variable. We analyzed by using statistical software, R version 4.5.2 (R Foundation for Statistical Computing, Vienna, Austria) and MatchIt version 4.7.2.

Table S1. Comparison of Clinical Characteristics in Propensity-Score Matched Patients with and without PolyVD

	PolyVD (-) (n=144)	PolyVD (+) (n=144)	P value	SMD
Age (years) *	71.0 ± 8.3	71.0 ± 9.3	0.995	0.001
Women, n (%)	30 (20.8)	29 (20.1)	1.000	0.017
BMI (kg/m^2) *	24.2 ± 3.3	24.0 ± 3.4	0.696	0.046
Hypertension, n (%)	114 (79.2)	124 (86.1)	0.161	0.184
Dyslipidemia, n (%)	121 (84.0)	118 (81.9)	0.754	0.055
Type 2 diabetes mellitus, n (%)	80 (55.6)	79 (54.9)	1.000	0.014
Current smoker, n (%)	21 (14.6)	28 (19.4)	0.347	0.130
CKD, n (%)	73 (50.7)	79 (54.9)	0.555	0.084
A history of MI, n (%)	38 (26.4)	29 (20.1)	0.265	0.148
ACS, n (%)	46 (31.9)	41 (28.5)	0.608	0.076
Statin, n (%)	110 (76.4)	106 (73.6)	0.683	0.064
High-intensity statin, n (%)	44 (30.6)	48 (33.3)	0.705	0.060
Ezetimibe, n (%)	30 (20.8)	36 (25.0)	0.483	0.099
LDL-C (mg/dL) †	90.0 [65.0, 114.0]	83.0 [61.8, 105.0]	0.112	0.181

Values are n (%), *mean ± standard deviation. †median (interquartile range).
ACS = acute coronary syndrome, BMI = body mass index, CKD = chronic kidney disease, LDL-C = low-density lipoprotein cholesterol, MI = myocardial infarction, PolyVD = polyvascular disease, SMD = standardized mean difference

Table S2. Comparison of Clinical Characteristics in Patients with PolyVD Diagnosed by History or Vascular Testing

	Patients with PolyVD (n=189)		p value
	Diagnosed by vascular testing (n=94)	Diagnosed by history (n=95)	
Age (years) *	75.0 [68.8-79.0]	74.0 [68.0-81.0]	0.995
Women, n (%)	21 (22.3)	13 (13.7)	0.121
BMI (kg/m^2) *	23.6 [21.7-25.1]	23.8 [21.8-25.6]	0.622
Hypertension, n (%)	69 (73.4)	91 (95.8)	<0.001
Dyslipidemia, n (%)	71 (75.5)	81 (85.3)	0.092
Type 2 diabetes mellitus, n (%)	52 (55.3)	47 (49.5)	0.421
Current smoker, n (%)	18 (19.1)	18 (18.9)	0.972
Family history, n (%)	13 (13.8)	23 (24.2)	0.069
CKD, n (%)	57 (60.6)	61 (64.2)	0.612
A history of MI, n (%)	20 (21.3)	21 (22.1)	0.890
ACS, n (%)	37 (39.4)	22 (23.2)	0.016
Medication Use on admission			
Statin, n (%)	65 (69.1)	78 (82.1)	0.038
High-intensity statin, n (%)	27 (28.7)	35 (36.8)	0.235
Ezetimibe, n (%)	22 (23.4)	25 (26.3)	0.643
Risk factor control			
LDL-C (mg/dL) *	86.0 [63.5-114.5]	79.0 [60.0-103.0]	0.115
HDL-C (mg/dL) *	45.0 [37.0-52.3]	47.0 [37.0-53.0]	0.431
Triglyceride (mg/dL) *	127.5 [86.5-165.3]	117.0 [81.0-171.0]	0.729
HbA1c (%) *	6.3 [5.7-6.9]	6.2 [5.8-6.7]	0.891
NIRS imaging			
$maxLCBI_{4mm}$ *	470 [319.5-726.3]	454.0 [354.0-698.0]	0.792
$maxLCBI_{4mm}$ ≥324.7	70 (74.5)	75 (78.9)	0.466

Values are n (%), * median (interquartile range).

ACS = acute coronary syndrome, BMI = body mass index, CKD = chronic kidney disease, HbA1c = glycated hemoglobin, HDL = high-density lipoprotein cholesterol, LDL-C = low-density lipoprotein cholesterol, $maxLCBI_{4mm}$ = maximum 4-mm lipid-core burden index, MI = myocardial infarction, PolyVD = polyvascular disease

Table S3. Comparison of NIRS-derived Measures in Propensity-Score Matched Cohort

(A)Patients without PolyVD (n=144)

	LDL-C			P value
	<55 mg/dL (n = 16)	55-69 mg/dL (n = 24)	≥70 mg/dL (n = 104)	
$maxLCBI_{4mm}$	222.5 [120.8-500.0]	484.5 [359.8-598.2]	558.5 [370.0-738.2]	0.031
$maxLCBI_{4mm}$ ≥324.7	6 (37.5)	19 (79.2)	86 (82.7)	0.001

(B)Patients with PolyVD (n=144)

	LDL-C			P value
	<55 mg/dL (n = 22)	55-69 mg/dL (n = 29)	≥70 mg/dL (n = 93)	
$maxLCBI_{4mm}$	431.0 [355.0-509.2]	422.0 [353.0-515.0]	562.0 [325.0-743.0]	0.481
$maxLCBI_{4mm}$ ≥324.7	17 (77.3)	23 (79.3)	70 (75.3)	0.747

Values are presented as median [interquartile range].
LDL-C = low-density lipoprotein cholesterol, $maxLCBI_{4mm}$ = maximum 4-mm lipid-core burden index, PolyVD = polyvascular disease
P for trend was calculated using weighted linear regression in the propensity score–matched cohort.

Table S4. Patients' Characteristics in Patients with and without PolyVD according to LDL-C Levels

	LDL-C								
	<55 mg/dL			55-69 mg/dL			≥70 mg/dL		
	PolyVD		P value	PolyVD		P value	PolyVD		P value
	- (n=22)	+ (n=29)		- (n=34)	+ (n=38)		- (n=177)	+ (n=122)	
Age (years) *	68.5 [62.2-77.5]	73.0 [65.5-81.0]	0.180	70 [58.7-77.0]	75.0 [66.5-79.0]	0.062	65.0 [57.0-73.0]	74.5 [69.0-79.0]	<0.001
Women, n (%)	4 (18.2)	4 (13.8)	0.713	7 (20.6)	4 (10.5)	0.329	37 (20.9)	26 (21.3)	0.932
BMI (kg/m^2) *	23.0 [19.8-26.8]	24.3 [21.7-25.5]	0.371	25.0 [21.8-27.1]	23.5 [22.1-26.4]	0.227	24.0 [22.0-25.8]	23.5 [21.4-25.3]	0.078
Hypertension, n (%)	18 (81.8)	25 (86.2)	0.713	26 (76.5)	31 (81.6)	0.594	124 (70.1)	104 (85.2)	0.002
Dyslipidemia, n (%)	20 (90.9)	24 (82.8)	0.684	30 (88.2)	31 (81.6)	0.522	150 (84.7)	97 (79.5)	0.240
Type 2 diabetes mellitus, n (%)	11 (50.0)	17 (58.6)	0.540	16 (47.1)	22 (57.9)	0.358	86 (48.6)	60 (49.2)	0.920
Current smoker, n (%)	2 (9.1)	2 (6.9)	1.000	1 (2.9)	6 (15.8)	0.111	34 (19.2)	28 (23.0)	0.433
Family history, n (%)	3 (13.6)	9 (31.0)	0.192	11 (32.4)	8 (21.1)	0.277	55 (31.1)	19 (15.6)	0.002
CKD, n (%)	8 (36.4)	23 (79.3)	0.002	15 (44.1)	21 (55.3)	0.345	66 (37.3)	74 (60.7)	<0.001
A history of MI, n (%)	11 (50.0)	12 (41.4)	0.540	12 (35.3)	13 (34.2)	0.923	36 (20.3)	16 (13.1)	0.105
ACS, n (%)	1 (4.5)	7 (24.1)	0.117	2 (5.9)	5 (13.2)	0.435	81 (45.8)	47 (38.5)	0.214
Lipid-lowering Therapies									
Statin, n (%)	22 (100.0)	27 (93.1)	0.500	34 (100.0)	37 (97.4)	1.000	116 (65.5)	79 (64.8)	0.889
High-intensity statin, n (%)	13 (59.1)	14 (48.3)	0.443	17 (50.0)	21 (55.3)	0.655	50 (28.2)	27 (22.1)	0.234
Ezetimibe, n (%)	11 (50.0)	11 (37.9)	0.389	15 (44.1)	16 (42.1)	0.863	30 (16.9)	20 (16.4)	0.899
PCSK9-I, n (%)	0 (0.0)	1 (3.4)	1.000	0 (0.0)	0 (0.0)	N/A	1 (0.6)	0 (0.0)	1.000
Aspirin, n (%)	21 (95.5)	26 (89.7)	0.625	31 (91.2)	32 (84.2)	0.485	168 (94.9)	117 (95.9)	0.786
$P2Y_{12}$ inhibitor, n (%)	21 (95.5)	29 (100.0)	0.431	34 (100.0)	38 (100.0)	N/A	176 (99.4)	122 (100.0)	1.000
ACEI/ARB, n (%)	14 (63.6)	18 (62.1)	0.909	20 (58.8)	22 (57.9)	0.936	75 (42.4)	65 (53.3)	0.063
β-blocker, n (%)	16 (72.7)	15 (51.7)	0.128	24 (70.6)	22 (57.9)	0.263	58 (32.8)	40 (32.8)	0.997
SGLT2i, n (%)	3 (13.6)	2 (6.9)	0.641	3 (8.8)	4 (10.5)	1.000	9 (5.1)	5 (4.1)	0.786
GLP-1 inhibitor, n (%)	0 (0.0)	3 (10.3)	0.249	2 (5.9)	4 (10.5)	0.677	10 (5.6)	9 (7.4)	0.547
Metformin, n (%)	4 (18.2)	3 (10.3)	0.447	7 (20.6)	5 (13.2)	0.530	25 (14.1)	16 (13.1)	0.803
Risk factor control									

LDL-C (mg/dL) *	49.0 [43.0-53.0]	49.0 [39.5-51.5]	0.188	63.0 [60.7-65.0]	62.5 [58.0-67.0]	0.786	107.0 [85.0-133.5]	98.5 [83.0-120.0]	0.035
HbA1c (%) *	6.5 [6.0-6.9]	6.3 [5.8-6.7]	0.198	6.1 [5.6-6.8]	6.4 [5.8-6.9]	0.278	6.2 [5.8-7.1]	6.1 [5.7-6.7]	0.242

Values are n (%), * median (interquartile range).
ACEI = angiotensin-converting enzyme inhibitor, ACS = acute coronary syndrome, ARB = angiotensin II receptor blocker, BMI = body mass index, CKD = chronic kidney disease, GLP-1 = Glucagon-like peptide-1, HbA1c = glycated hemoglobin, LDL-C = low-density lipoprotein cholesterol, MI = myocardial infarction, PCSK9-I = proprotein convertase subtilisin/kexin type 9 inhibitor, PolyVD = polyvascular disease, SGLT2i = Sodium-Glucose cotransporter 2 inhibitor

Figure S1. Patients' Disposition

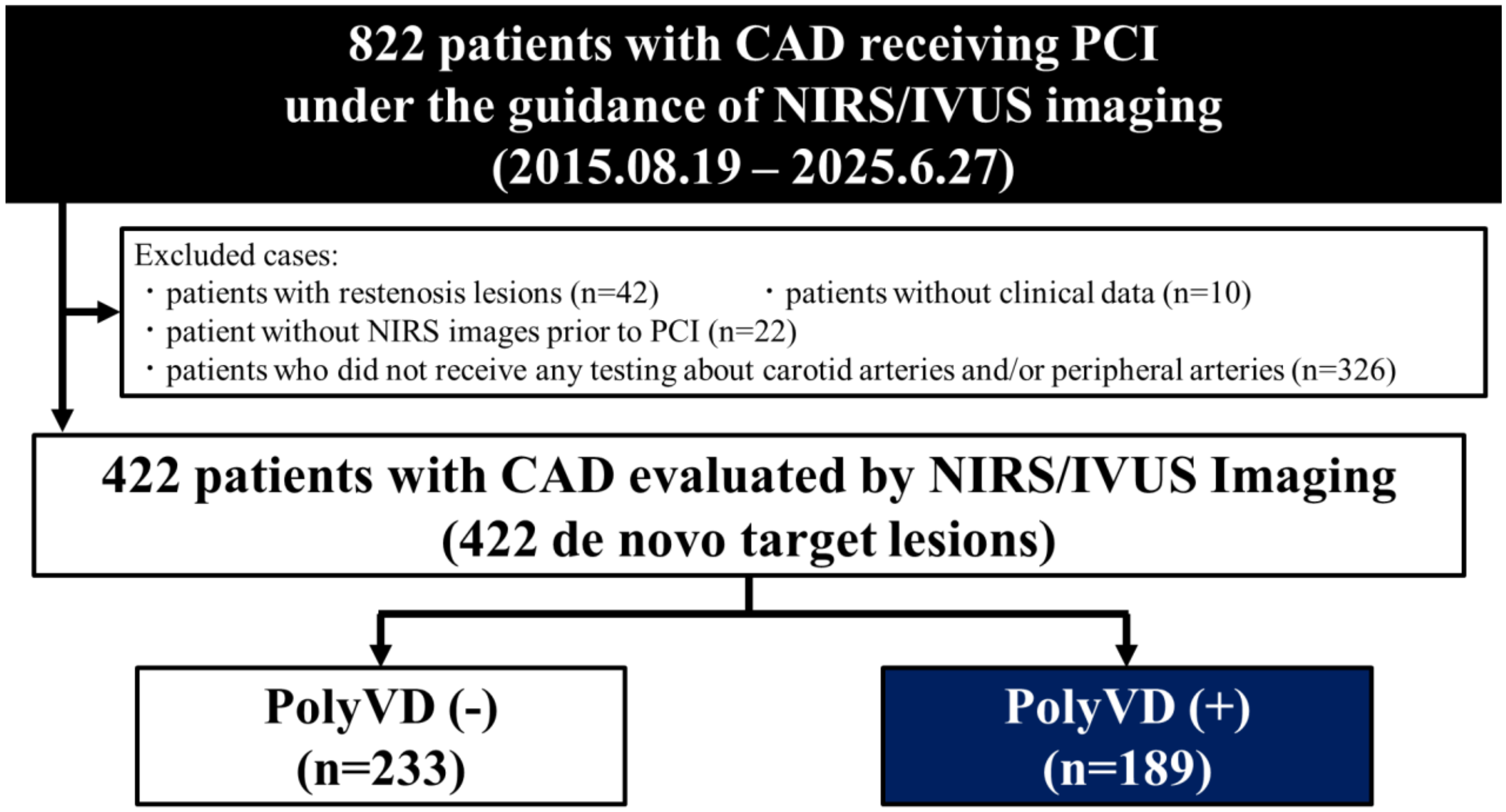

CAD = coronary artery disease, NIRS/IVUS = near-infrared spectroscopy and intravascular ultrasound, PCI = percutaneous coronary intervention, PolyVD = polyvascular disease

Figure S2. Propensity Score Matching of Patients with and without PolyVD

(A) Covariate Balance Before and After Propensity Score Matching

(B) Distribution of Propensity Scores Before and After Matching

(A) **(B)**

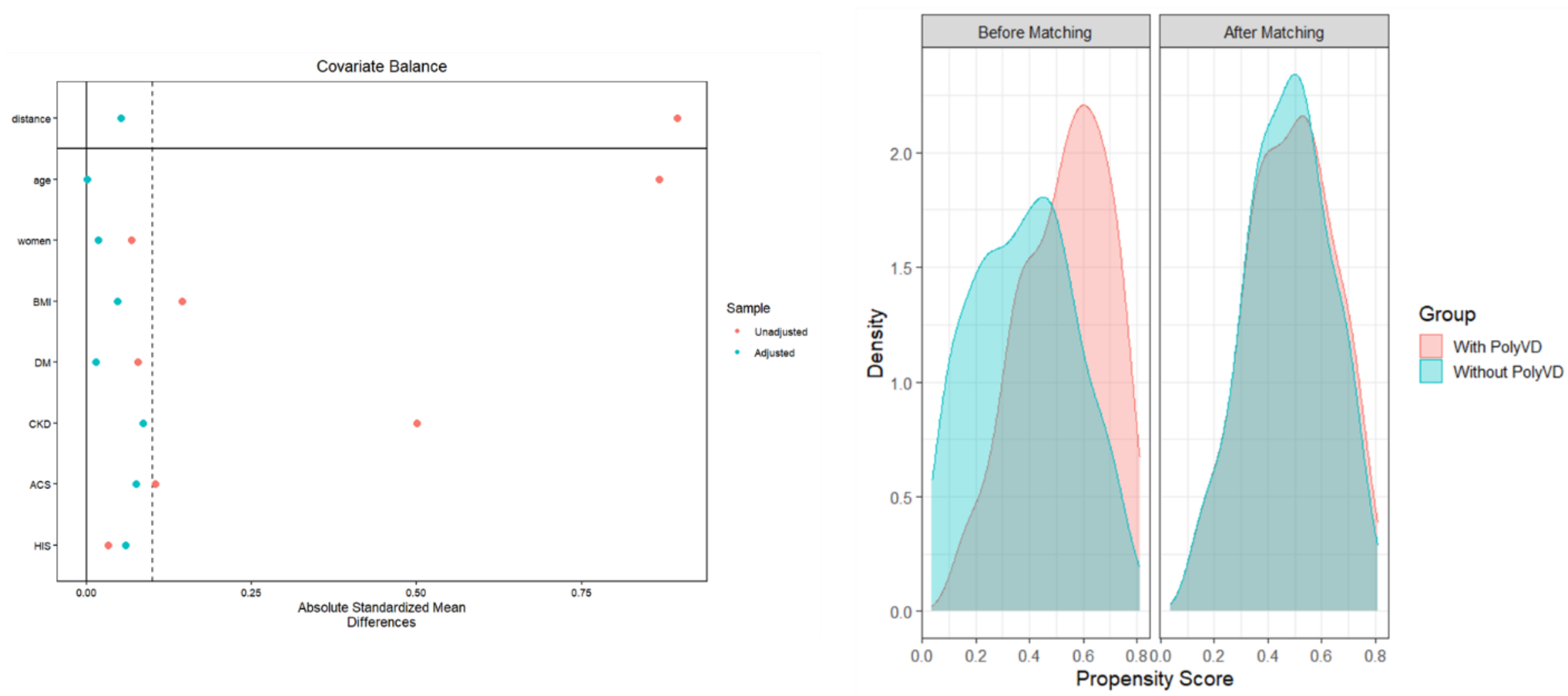

(A) The covariate balance plot (Love plot) depicts the absolute standardized mean differences for baseline covariates before (unadjusted) and after (adjusted) propensity score matching. After matching, the differences across all covariates were reduced to below the conventional balance threshold of 0.1 (vertical dashed line).
ACS = acute coronary syndrome, BMI = body mass index, CKD = chronic kidney disease, DM = diabetes mellitus, HIS = high intensity statin
(B) The density plots depict the distribution of propensity scores in patients with and without PolyVD group before and after matching. The substantial overlap in the distributions after matching suggests that propensity score matching yielded a well-balanced cohort for the present analysis.
PolyVD = polyvascular disease
